# Antibiotic Prescribing Habits of Dental Surgeons in Hyderabad City, India, for Pulpal and Periapical Pathologies: A Survey

**DOI:** 10.1155/2013/537385

**Published:** 2013-09-26

**Authors:** K. Pavan Kumar, Mamta Kaushik, P. Udaya Kumar, M. Shilpa Reddy, Neha Prashar

**Affiliations:** Department of Conservative Dentistry and Endodontics, Army College of Dental Sciences, Secunderabad 500087, India

## Abstract

*Aim*. To determine the antibiotic prescribing habits for pulpal and periapical pathology among dentists in Hyderabad city, India. *Methodology*. A total of 246 questionnaires were distributed to all the dentists registered with the local dental branch. Demographic details and questions regarding type and dosage of antibiotics prescribed for allergic and nonallergic patients were recorded. Inferential statistics were performed, and *P* < 0.05 was considered statistically significant. *Results*. The response rate for the study was 87.8%. Around 148 (68.5%) of respondents regularly prescribed antibiotics for endodontic management. The first antibiotic of choice for patients with no history of medical allergies was a combination of amoxicillin and metronidazole, followed by amoxicillin alone (29.1%). The first antibiotic of choice in case of allergy to penicillin was erythromycin. Necrotic pulp with acute apical periodontitis with swelling and moderate/severe preoperative symptom was the condition most commonly identified for antibiotic therapy (92.1%). *Conclusion*. The present study reveals that the overall antibiotic prescribing practices among this group of dentists were quite high, and there is a need for more educational initiatives to rationalize the use of antibiotics in dentistry.

## 1. Introduction

In the health care industry, the advent of antibiotics constitutes one of the greatest revolutionary advancements. Dental infections are polymicrobial involving a combination of gram positive, gram negative, facultative anaerobes, and strict anaerobic bacteria [1]. Thus, antibiotics and analgesics account for a vast majority of medicines prescribed by the dentists.

Penicillin and other antibiotics, which were initially viewed as miracle drugs for their ability to cure serious and often life-threatening diseases, were challenged by some defiant strains. Antibiotic resistance has become a serious public health concern. Reasons for the development of antimicrobial resistance could be due to overprescription by health care providers and improper use by patients [2].

In endodontics, it is recommended that antibiotics should be used only as an adjunct to definitive nonsurgical or surgical endodontic therapy [3]. Despite this, use of antibiotics has been observed on a regular basis in dental practice [3–8]. The literature review reveals that in India, there is no established pattern for prescription of antibiotic for various endodontic pathologies. The present study was aimed to determine the antibiotic prescribing practices for pulpal and periapical pathology among dentists in Hyderabad city, India.

## 2. Materials and Method

A cross-sectional survey was designed to determine the antibiotic prescribing habits amongst dentists of Hyderabad city, AP, India. A total of 246 questionnaires were distributed to all the dentists registered with the local dental association branch. Demographic details and questions regarding type and dosage of antibiotics prescribed for allergic and nonallergic patients were recorded. Data were computed and analyzed using SPSS software (version 12.00). Statistical analysis was done using chi-square test, and *P* < 0.05 was considered statistically significant.

Ethical Committee Clearance from the Institutional Review Board was obtained.

## 3. Results

Of the 246 questionnaires distributed, 216 were returned completely filled (response rate: 87.8%).  Table 1 represents the demographic details of the respondents based on gender. Mean age of the respondents was 28.6 ± 6.5 years. Majority of them belonged to the age group of 21–25 years (42.1%). 60.5% of the respondents possessed bachelor's degree (Bachelor of Dental Surgery—BDS) and 39.5% of the respondents possessed master's degree (Master of Dental Surgery—BDS). 

148 (68.5%) of respondents regularly prescribed antibiotics for endodontic management. This was not significant when compared with those who did not advise (*P* = 0.5). Though it was noted that a variety of antibiotic combinations were recommended, the most common for patients with no history of medical allergies was a combination of amoxicillin 500 mg with metronidazole 400 mg, twice a day followed by amoxicillin 500 mg alone (29.1%) (Table 2). 

Around 51.8% of this study population prescribed antibiotics for a duration of five days, and only five respondents (2.31%) have stated to prescribe it for seven days. A statistically significant difference was noted when comparison of age groups with the number of days of antibiotic prescription was done (*P* = 0.04) with more of elder age group recommending for five days. 

The first antibiotic of choice in case of allergy to penicillin was erythromycin 250 mg, three times a day prescribed by 53.7% of the respondents (Table 3).

Table 4 demonstrates the various endodontic conditions necessitating antibiotic prescription. Necrotic pulp with acute apical periodontitis with swelling and moderate/severe preoperative symptom was the most commonly identified condition for antibiotic therapy (92.1%). This was then followed by necrotic pulp with chronic apical periodontitis and sinus tract with no/mild preoperative symptoms (69.4%). The cases with necrotic pulp with chronic apical periodontitis with no swelling and no/mild preoperative symptoms were identified as the condition receiving least antibiotic prescription (44.9%).

## 4. Discussion

The present survey evaluated the antibiotic prescribing practices for endodontic pathologies amongst dentists in Hyderabad city, India. The response rate for the study was 87.8%, which was considered acceptable. 

Our study noted that around 68.5% of the population regularly prescribed antibiotics for endodontic management with 51.8% prescribing them for five days. On comparison with other countries, it was observed that in 2008 in the United Kingdom [9], 40% of the dentists prescribe antibiotics. In Belgium in 2009, antibiotics were prescribed by dentists to only 4.2% of the population [10]. In a survey in Canada [11] in 2000, it was found that on average, duration of antibiotics prescribed by dentists was 6.92 days. Likewise, Yingling et al. [12] reported that in the United States in 2002, endodontists prescribe antibiotics for an average of 7.58 days. Duration of antibiotic prescription has always been variable. Short course of antibiotic usage, for two-three days has shown an improvement in patient condition [13–16] and is usually preferred in children for better compliance [17].

In patients with no history of medical allergies, Indian dentists largely prescribed a combination of amoxicillin and metronidazole (30%). The rationale for the choice of amoxicillin could have been its wide spectrum, low incidence of resistance, pharmacokinetic profile, tolerance, and dosage [18, 19]; combination with metronidazole enhances the anaerobic activity. On the other hand, studies on Lithuanian [8], Spanish [20], and American [12] dentists revealed amoxicillin as the first drug of choice for orofacial infections.

In case of allergy to penicillin, erythromycin was regarded as the drug of choice by 54.6% of the respondents. This could be attributed to the fact that erythromycin has similar spectrum of activity as amoxicillin.

Though endodontic conditions like irreversible pulpitis with moderate/severe preoperative symptoms with or without acute periodontitis do not warrant antibiotic coverage, in the present study 60.6% and 65.2% respondents prescribed antibiotics. This finding is slightly higher than those reported in the previous studies [5, 6, 8, 12]. Necrotic pulp with chronic apical periodontitis with no swelling and no/mild symptoms also has no indication of antibiotic use as treatment can be restricted to nonsurgical root canal therapy; however, 44.9% of this study population reported antibiotic use. This result was higher as compared to the survey done by Rodriquez-Núñez et al. [20]. Around 56.9% of the survey population prescribed antibiotics for cases with necrotic pulp with acute apical periodontitis, no swelling, and moderate/severe preop. symptoms, which can effectively treated with root canal therapy and analgesics. This finding is comparable with the studies by Rodriquez-Núñez et al. [20] (52.9%) and Yingling et al. [12] (53.9%).

In the present study a higher percentage of dentists prescribed antibiotics for necrotic pulp with chronic apical periodontitis with sinus tract and no/mild preop. symptoms as compared to Spanish [20] and American [12] dentists. The condition of necrotic pulp with acute apical periodontitis with swelling and moderate/severe preop. symptoms usually indicates an antibiotic coverage which was agreed upon by around 92% of the respondents. Comparison with previous studies [12, 20–22] revealed similar finding between 87.6% to 99.2%.

In endodontics, dental practitioners are dealing with a very small number of extremely virulent bacteria. A majority of these can be dealt with by the most common of antibiotics. Problems may arise from prescriptions that are issued for an inappropriate antibiotic, in inappropriate circumstances at inadequate daily dosage, and with no initial loading dose.

## 5. Conclusion

The present study reveals that overall the antibiotic prescribing practices among this group of dentists were quite high. This study emphasizes the need for more educational initiatives to rationalize the use of antibiotics in dental practice. Also, prescription of antibiotics should be at the correct dosage and duration to prevent the development of resistant bacteria. 

## Figures and Tables

**Table 1:** Description of the respondents based on gender and academic qualification.

Variable	Gender		*N* (%)
	Male *N* (%)	Female *N* (%)	Total *N* (%)
Age group			
21–25 yrs	29 (31.9%)	62 (68.1%)	91 (42.1%)
26–30 yrs	29 (46.1%)	34 (53.9%)	63 (29.2%)
31–35 yrs	23 (85.2%)	4 (14.8%)	27 (12.5%)
36–40 yrs	17 (85%)	3 (15%)	20 (9.3%)
41+ yrs	10 (66.7%)	5 (33.3%)	15 (6.9%)
Total	**108 (50%)**	**108 (50%)**	**216 (100%)**

Academic degree			
BDS	53 (40.5%)	78 (59.5%)	131 (60.5%)
MDS	55 (64.7%)	30 (35.3%)	85 (39.5%)
Total	**108 (50%)**	**108 (50%)**	**216 (100%)**

**Table 2:** Type, dose, and frequency of antibiotic prescription for an adult patient with no allergy to penicillin.

Which antibiotic do you prescribe most often for adult patient with no medical allergies?	bd *N* (%)	tds *N* (%)	Total *N* (%)
Amoxicillin 500 mg	11 (17.5%)	52 (82.54%)	63 (29.3%)
Amoxicillin + clavulanic acid 625 mg	22 (50%)	22 (50%)	44 (20.5%)
Amoxicillin 250 mg	3 (75%)	1 (25%)	4 (1.9%)
Amoxicillin 250 mg + cloxacillin 250 mg	0 (0%)	1 (100%)	1 (0.4%)
Amoxicillin 250 mg + metronidazole 400 mg	0 (0%)	1 (100%)	1 (0.4%)
Amoxicillin 500 mg + metronidazole 400 mg	41 (63.1%)	24 (36.9%)	65 (30.2%)
Amoxicillin + cloxacillin 500 mg	5 (62.5%)	3 (37.5%)	8 (3.8%)
Amoxicillin + clavulanic acid 625 mg + metronidazole 400 mg	1 (50%)	1 (50%)	2 (0.9%)
Amoxicillin + clavulanic acid 375 Mg	0 (0%)	1 (100%)	1 (0.4%)
Amoxicillin + clavulanic acid 500 mg	1 (100%)	0 (0%)	1 (0.4%)
Amoxicillin + cloxacillin 500 mg + metronidazole 400 mg	1 (100%)	0 (0%)	1 (0.4%)
Cefadroxil 500 mg	1 (100%)	0 (0%)	1 (0.4%)
Ciprofloxacin 500 mg + tinidazole 300 mg	1 (100%)	0 (0%)	1 (0.4%)
Erythromycin 500 mg	2 (100%)	0 (0%)	2 (0.9%)
Metronidazole 400 mg	0 (0%)	2 (100%)	2 (0.9%)
Ofloxacillin 200 mg + ornidazole 500 mg	18 (94.7%)	1 (5.3%)	19 (8.9%)

Grand total	107 (49.5%)	109 (50.5%)	216 (100%)

**Table 3:** Type, dose, and frequency of antibiotic prescription for an adult patient with allergy to penicillin.

Which antibiotic do you prescribe most often?	bd *N* (%)	od *N* (%)	qid *N* (%)	tds *N* (%)	Total *N* (%)
Azithromycin 500 mg	0 (0%)	5 (83.3%)	0 (0%)	1 (16.7%)	6 (2.8%)
Cefadroxil 500 mg	1 (100%)	0 (0%)	0 (0%)	0 (0%)	1 (0.4%)
Cefixime 200 mg	8 (88.9%)	1 (11.1%)	0 (0%)	0 (0%)	9 (4.3%)
Cephalexin 500 mg	14 (100%)	0 (0%)	0 (0%)	0 (0%)	14 (6.5%)
Ciprofloxacin 200 mg	1 (100%)	0 (0%)	0 (0%)	0 (0%)	1 (0.4%)
Ciprofloxacin 500 mg	21 (91.3%)	0 (0%)	0 (0%)	2 (8.7%)	23 (10.6%)
Ciprofloxacin 500 mg + tinidazole 600 mg	2 (100%)	0 (0%)	0 (0%)	0 (0%)	2 (0.9%)
Clindamycin 300 Mg	6 (100%)	0 (0%)	0 (0%)	0 (0%)	6 (2.8%)
Doxycyclin 100 mg	5 (83.3%)	1 (16.7%)	0 (0%)	0 (0%)	6 (2.8%)
Erythromycin 500 mg	55 (46.7%)	0 (0%)	2 (1.6%)	61 (51.7%)	118 (54.6%)
Levofloxacin 500 mg	0 (0%)	1 (100%)	0 (0%)	0 (0%)	1 (0.4%)
Metronidazole 400 mg	8 (88.9%)	0 (0%)	0 (0%)	1 (11.1%)	9 (4.3%)
Ofloxacillin 200 mg	4 (100%)	0 (0%)	0 (0%)	0 (0%)	4 (1.9%)
Ofloxacillin 200 mg + ornidazole 500 mg	10 (83.3%)	0 (0%)	0 (0%)	2 (16.7%)	12 (5.6%)
Ofloxacin 400 mg	2 (100%)	0 (0%)	0 (0%)	0 (0%)	2 (0.9%)
Ornidazole 500 mg	1 (100%)	0 (0%)	0 (0%)	0 (0%)	1 (0.4%)
Tetracycline 500 mg	1 (100%)	0 (0%)	0 (0%)	0 (0%)	1 (0.4%)

Total	139 (64.4%)	8 (3.7%)	2 (0.9%)	67 (31%)	216 (100%)

**Table 4:** Endodontic conditions necessitating antibiotics prescription.

Conditions	Number of respondents *N* (%)
(a) Irreversible pulpitis, mod/severe preop. symptoms	131 (60.6%)
(b) Irreversible pulpitis with acute apical periodontitis, mod/severe preop. symptoms	141 (65.2%)
(c) Necrotic pulp with chronic apical periodontitis, no swelling, no/mild preop. symptoms	97 (44.9%)
(d) Necrotic pulp with acute apical periodontitis, no swelling, mod/severe preop. symptoms	123 (56.9%)
(e) Necrotic pulp with chronic apical periodontitis, sinus tract present, no/mild preop. symptoms	150 (69.4%)
(f) Necrotic pulp with acute apical periodontitis, swelling present, mod/severe preop. symptoms	199 (92.1%)
